# Antimicrobial Coating Efficacy for Prevention of *Pseudomonas aeruginosa* Biofilm Growth on ISS Water System Materials

**DOI:** 10.3389/fmicb.2022.874236

**Published:** 2022-04-07

**Authors:** Madelyn K. Mettler, Ceth W. Parker, Kasthuri Venkateswaran, Brent M. Peyton

**Affiliations:** ^1^Center for Biofilm Engineering, Montana State University, Bozeman, MT, United States; ^2^NASA Jet Propulsion Laboratory, California Institute of Technology, Pasadena, CA, United States

**Keywords:** biofilm, *Pseudomonas aeruginosa* PA14, antimicrobial, International Space Station, NASA, built environment, CDC reactor

## Abstract

Biofilms can lead to biofouling, microbially induced corrosion, physical impediment and eventual loss in function of water systems, and other engineered systems. The remoteness and closed environment of the International Space Station (ISS) make it vulnerable to unchecked biofilm growth; thus, biofilm mitigation strategies are crucial for current ISS operation and future long duration and deep-space crewed missions. In this study, a space flown bacterial strain of *Pseudomonas aeruginosa* (PA14) was used as a model organism for its ability to form biofilms. Additionally, a novel antimicrobial coating’s ability to reduce biofilm accumulation on stainless steel, Teflon, titanium, and Inconel (all used in the ISS water treatment and handling systems) was analyzed. Coated materials demonstrated reductions of *P. aeruginosa* biofilm across all materials when tested in a continuous flow system with tryptic soy broth medium. However, the coating lost efficacy in potato dextrose broth medium. These findings were corroborated *via* scanning electron microscopy. This study illustrates the fundamental importance of using multiple approaches to test antibiofilm strategies, as well as the specificity in which conditions such strategies can be implemented.

## Introduction

Biofilms are complex interconnected communities of surface attached biological materials containing the majority of microorganisms on Earth (Wingender and Flemming, 2011). Biofilms confer many advantages to their constituent microorganisms over planktonic cells, including increased resistance to antimicrobials and environmental stresses, easier metabolite interchange (Santos et al., 2018), and increased genetic material exchange *via* horizontal gene and plasmid transfer (Madsen et al., 2012). These characteristics impart survival advantages to biofilm communities making their control more difficult than planktonic microorganisms.

Along with medical implant rejection, catheter infections, and tooth decay (Aparna and Yadav, 2008), biofilms negatively impact society through biofouling (Bixler and Bhushan, 2012). Biofouling is the colonization of any solid material by microorganisms that increases the corrosion rate, fluid drag, and subsequent system contamination (Characklis and Cooksey, 1983) with many built environments susceptible to biofouling (Gule et al., 2016). Bacteria and other microorganisms can increase the rate and extent of corrosion, causing microbial-induced corrosion (MIC; Kitsutani et al., 1970). This MIC of metal surfaces results from interactions between susceptible surfaces and microbial products including acids, volatile compounds, and electrons (Beech and Sunner, 2004). Biofilms, biofouling, and MIC have a large economic impact, with estimated global cost above $1 billion annually (Rajala et al., 2015; Little et al., 2020).

While biofilms are ubiquitous on Earth (Flemming and Wuertz, 2019), their reach extends into space in microgravity environments of human-made spacecraft. The decommissioned Mir space station was heavily contaminated with biofilms (Klintworth et al., 1999; Kim et al., 2013), and biofouling aboard the International Space Station (ISS) resulted in obstruction of the water processing assembly (Carter, 2009). The microgravity environment can lead to increased production of extracellular polymeric substances, virulence, antimicrobial resistance, shorter lag and longer exponential phases of microorganisms, and biofilms (Klaus et al., 1997; Nickerson et al., 2004; Lynch et al., 2006; Mauclaire and Egli, 2010; Rosenzweig et al., 2010; Searles et al., 2011). The traditional cleaning methods of disassembly, harsh chemicals, purging water supplies, and/or replacing compromised hardware to mitigate biofilm growth are incredibly difficult (and sometimes impossible) in a confined spacecraft in microgravity. These issues will become more exacerbated as the National Aeronautics and Space Administration (NASA) begins longer missions to send humans to the Moon and Mars, making resupply and replacement parts more costly and harder to execute. Engineers have begun to design and construct the Lunar Gateway and next-generation Lunar landers; thus, it is critical that antibiofilm technologies be tested and successfully incorporated into deep-space habitats.

NASA has ongoing interest in mitigating biofilm growth to improve the viability of long-term space missions. This project sought to evaluate the four materials most used in the ISS water treatment system (stainless steel, Teflon, titanium, and Inconel) and thus most at risk for detrimental biofilm accumulation (Callahan et al., 2007). These materials are also being considered as primary components for constructing NASA’s Lunar Gateway water treatment and processing systems. The materials are also common in other parts of the craft. The objectives of this study were to characterize and quantify biofilm formation using a model bacterium in a continuous flow system using the Center for Disease Control (CDC) bioreactor and test the efficacy of a biocidal coating to prevent or reduce biofilm accumulation. The coating is a patented organosilane, surface penetrating formula. In the presence of hydroxyl groups at the surface of glass, minerals, or metals (e.g., aluminum and steel), silanols will form a stable Si bond. This is the key chemistry that allows silanes to function as valuable surface-treating/protecting or “coupling” agents. Four materials used in the ISS were challenged with the known biofilm forming model organism, *Pseudomonas aeruginosa* (previously space flown strain PA14). *P. aeruginosa* has been very well studied in biofilm systems and is a good benchmark for comparison to other research (Klintworth et al., 1999; Kim et al., 2013). This study will be useful for developing countermeasures to safeguard current and future spacecraft and their inhabitants.

## Materials and Methods

### Microorganisms Used

Pure culture of reference strain *P. aeruginosa* PA14 was obtained from Luis Zea at the BioServe Space Technologies (University of Colorado Boulder, United States). This bacterium is a Gram-negative, motile, and facultative aerobe. *P. aeruginosa* PA14 is a strain that has been previously flown to and cultured on the ISS in other experiments (Kim et al., 2013). Separate vials of stock cultures were maintained in glycerol at –80°C to ensure a reproducible inoculum throughout the experiment.

### CDC Bioreactor Preparation

**Figure 1:**
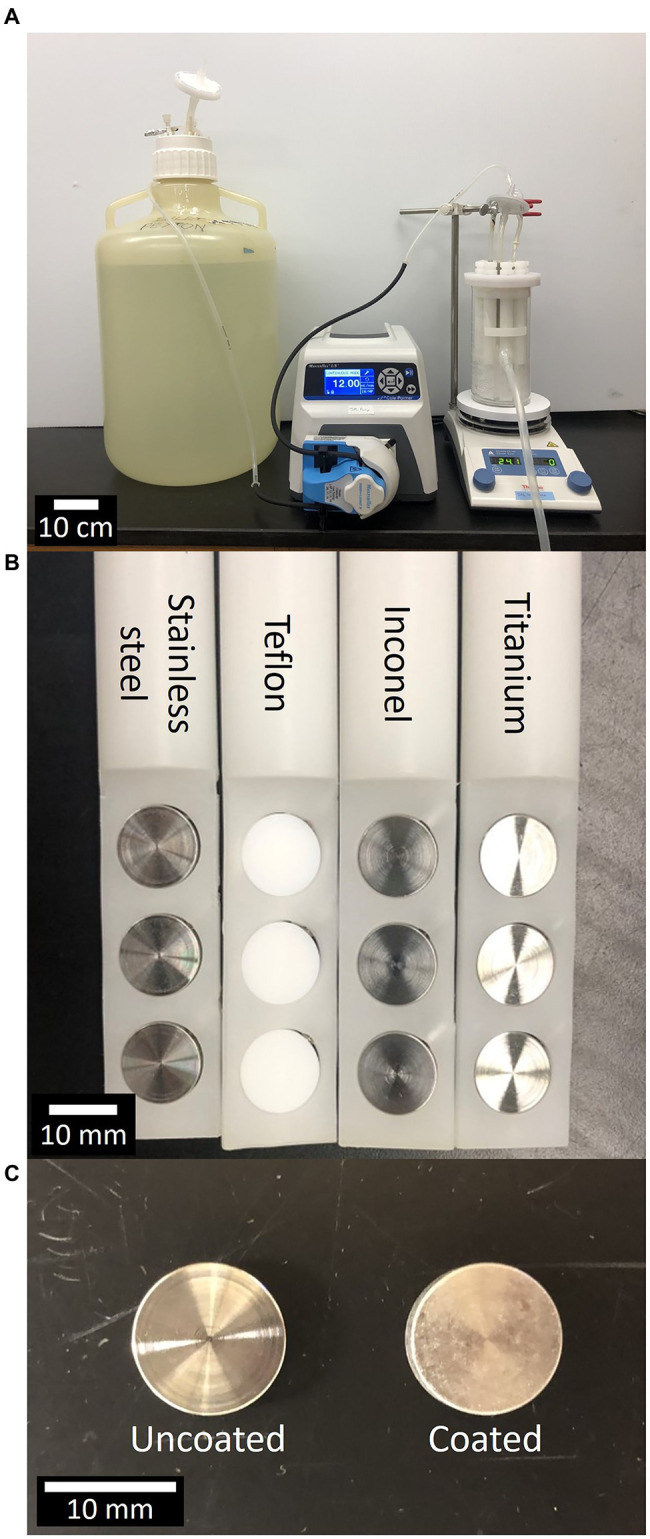
Center for Disease Control (CDC) bioreactor, material types, uncoated vs. coated materials. **(A)** Experimental setup consisting of 20 L carboy, peristaltic pump, and CDC Biofilm Reactor atop a stir plate. **(B)** Close-up of materials loaded into CDC Biofilm Reactor rods, from left to right, materials are stainless steel, Teflon, Inconel, and titanium. **(C)** Close-up of two titanium materials without coating (left) and with antimicrobial coating (right).

The experiments were conducted in CDC biofilm reactors (BioSurface Technologies, Bozeman, MT) in a continuous flow system (Figure 1A) The ISS materials evaluated were purchased from a commercial vendor (BioSurface Technologies) and consisted of 12.7 mm diameter cylindrical coupons of 316 L stainless steel, Teflon, Inconel 718 (a nickel-chromium alloy), and Ti-6AI4V titanium (Figure 1B). The ISS materials were purchased and precision cleaned at the Jet Propulsion Laboratory (JPL) following NASA standard practices developed for cleaning spacecraft components as previously described in Venkateswaran et al. (2004). Briefly, cleaning with a water-based detergent was followed by solvent immersion with ultrasonic agitation, prior to application of antibiofilm coating, and/or use in the CDC bioreactor. Once precision cleaned, the materials were sent to a commercial vendor for application of the antimicrobial coating (Figure 1C).

The CDC reactor, including the rods without ISS materials, was assembled and autoclaved empty (121°C and 20 PSI for 20 min) to ensure sterility. In a biosafety cabinet, the ISS materials were aseptically loaded into rods which were then submerged in isopropanol (IPA, 99%) for approximately 30 s, allowed to air dry for an additional 30 s, and replaced in the reactor. One trial run was performed using coated materials without isopropanol sterilization to ensure the sterilization process did not affect the efficacy of the coating. It was found that the materials sterilized by submersion in IPA fostered less microbial accumulation than coated materials that were not sterilized prior to use in the reactor (data not shown). All four materials were evaluated in triplicate simultaneously in the same reactor with each rod holding three coupons of the same material type. When uncoated and coated materials were initially tested together, the uncoated materials showed a reduction in cell density compared to uncoated materials tested alone. Further, coated materials tested in the same reactor with uncoated materials experienced an increase in cell density compared to coated materials tested alone (data not shown). Therefore, the remaining experiments (data presented in this paper) were completed with uncoated and coated materials tested in separate CDC bioreactors.

### Microorganism Preparation for CDC Bioreactor

*P. aeruginosa* was grown on tryptic soy agar (TSA) and incubated for 24 ± 2 h at 37°C to isolate individual colonies. Two colonies were used to inoculate a flask containing 100 ml of 300 mg/L TSB or 100 ml of 24 g/L PDB. The *P. aeruginosa* inoculum flasks were placed in an incubator-shaker at 150 rpm and 37°C for 24 ± 2 h and the colony forming units (CFU) were counted (TSB: 2.71 × 10^8^ ± 1.75 × 10^8^ CFU/ml, PDB: 1.01 × 10^9^ ± 6.43 × 10^8^ CFU/ml, average ± SD). To inoculate the CDC reactor, 1 ml of this inoculum was aseptically introduced to approximately 320 ml of sterile liquid medium such that initial cell counts in the reactor were approximately two orders of magnitude less than the cell counts of the inoculum flasks.

### Biofilm Growth

The method for operating the CDC biofilm reactors and growing the biofilms was based on the ASTM Standard Practice E31610-18 for preparing a *P. aeruginosa* or *Staphylococcus aureus* biofilm using the CDC Biofilm Reactor (ASTM International, 2018). Sterile liquid medium consisting of 300 mg/L TSB or 24 g/L PDB was added to the sterile reactor, and as described above, 1 ml of inoculum was introduced through the inoculation port on the CDC reactor lid. The reactor was placed on a stir plate at 125 rpm and operated in batch mode with no flow for 24 ± 2 h at room temperature. The reactors were operated in batch mode to allow cells to attach to the surfaces of the reactor and test materials. At *t* = 0 h, a continuous flow of fresh sterile medium of 100 mg/L TSB or 600 mg/L PDB was introduced to the reactor at a flow rate to create a liquid residence time of 30 min. The volumetric flow rate of the influent medium was sufficiently high (dilution rate greater than maximum specific growth rate) to wash planktonic cells out of the reactor before they could replicate, while providing fresh medium to the attached biofilm cells. Across all three reactors used, flow rate was set to 10.67 ± 0.12 ml/min (average ± SD).

### CDC Bioreactor Sampling

Planktonic cells were sampled from the reactor liquid at the start of continuous flow (*t* = 0 h) and at various sampling time points. Approximately 5 ml of reactor broth (bulk fluid) was removed with a serological pipette from an empty reactor rod slot. Due to the hydromechanics of continuously stirred tank reactors such as this one, the effluent media concentrations and cell densities are the same as in the bulk fluid. Planktonic cell samples were vortex mixed for 30 s prior to serial dilution and measured as CFU on TSA. Plates were incubated at 37°C for 24 ± 2 h before counting CFU.

For biofilm measurements, rods were removed at 24 ± 2 h and 48 ± 2 h after the start of continuous flow, gently rinsed in 30 ml of 0.01 M sterile phosphate buffered saline (PBS; 0.01 M PO_4_^=^; 0.137 M NaCl; and 0.0027 M KCl, adjusted to pH 7.4 at 25°C) to remove loosely attached cells, and test materials were placed into 50 ml sterile conical vials containing 10 ml of sterile PBS. The removed rods were replaced with sterile “dummy” rods to avoid altering the fluid dynamics of the reactor. Biofilm was disaggregated *via* an alternating vortex and sonication series of 30 s intervals beginning and ending with vortex mixing for a total of 2 min and 30 s. Disaggregated biofilm was serially diluted in PBS and plated on TSA for enumeration.

### Statistical Analysis

Statistical analysis of biofilm and planktonic cell counts from the CDC biofilm reactor sampling was carried out using MiniTab 19.2 (MiniTab, LLC). Statistical significance was determined *via* mixed effects ANOVA to determine the effects of the sampling time, experiment number (replicate), and material type on log reduction. The biofilm and planktonic cells were enumerated *via* CFU. The CFUs were converted to log densities for statistical evaluation. The ability of the coating and materials to reduce biofilm growth was evaluated on the basis of log reductions. To calculate the log reduction, the log density of the treatment experiment (coating) was subtracted from the log density of the control experiment. A negative log reduction indicates the coating resulted in increased CFUs. Similar calculations were made for planktonic cell growth.

### Scanning Electron Microscopy

The materials tested were preserved using 4% paraformaldehyde (PFA) prepared in 0.1 M PBS for scanning electron microscopy (Danko et al., 2020). Following the rinse step after removal from the reactor, coupons were fully immersed in a chilled 4% PFA and incubated at 4°C for 10 min and then, PFA was removed. The test material was rinsed thrice with chilled 0.1 M PBS and incubated at 4°C for 10 min between each rinse. After the third rinse, the vial with test material was filled completely with 0.1 M PBS and stored at 4°C until evaluated for scanning electron microscopy (SEM).

Biofilms grown in TSB and uninoculated coupons were imaged at the JPL. Fixed test coupons were transferred into a 24 well uncoated plate (Corning Inc., Corning, NY, United States) and dehydrated in IPA series with increasing IPA% as follows: 50%, 70%, 80%, 90%, 95%, and 100%, with the final 100% IPA replaced three times for 10 min each. Dehydrated test materials were stored at 4°C. The samples were then dried in a Tousimis Automegasamdri 915B critical point dryer (Rockville, MD, United States). Samples were adhered with carbon tape to SEM (Danko et al., 2020) stubs (Ted Pella Inc., Redding, CA, United States) and sputter coated using a Gold–Palladium target with an Anatech Hummer (Sparks, NV, United States) sputterer. A FEI Quanta 200F microscope (Thermo Fisher, Waltham, MA, United States) was used to capture SEM micrographs.

Unprocessed coupons and PDB biofilms were imaged at the Center for Biofilm Engineering (CBE) with a Zeiss SUPRA 55VP field emission SEM (Jena, Germany). Coupons with biofilm were critical point dried using a Tousimis SAMDRI-795. Prior to drying, the fixed coupons went through the same dehydration series as described above; however, ethanol was used rather than IPA due to the requirements by the CBE critical point dryer. The Teflon surfaces were sputter coated using an Iridium target with a Pella 108 Carbon Coater (Ted Pella Inc., Redding, CA, United States) prior to imaging.

## Results

### Biofilm Measurement Using Continuous Flow CDC Bioreactor

**Figure 2:**
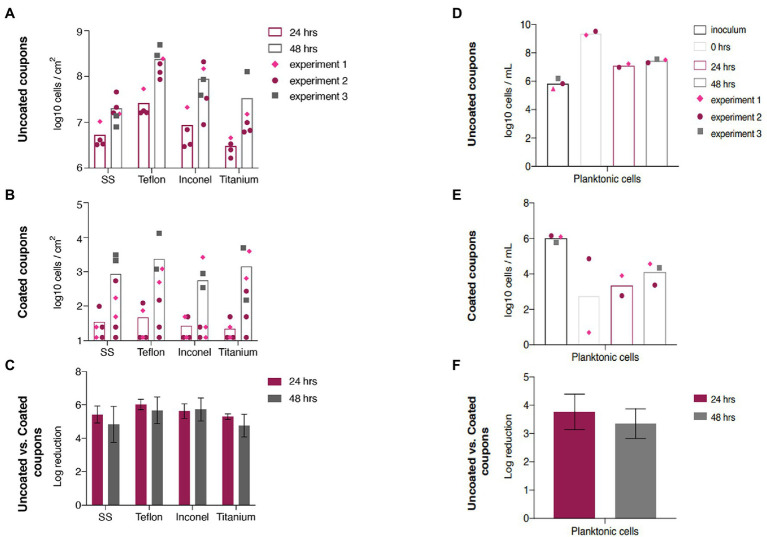
Tryptic soy broth (TSB)-grown *Pseudomonas aeruginosa* biofilm accumulation and planktonic cell density. TSB-grown *P. aeruginosa* biofilm cell densities at 24 and 48 h after start of continuous flow on **(A)** uncoated and **(B)** coated materials. **(C)** Log reduction of TSB-grown biofilm accumulation on materials at 24 and 48 h. Inoculum and planktonic cell densities at 0, 24, and 48 h after start of continuous flow for reactors with **(D)** uncoated and **(E)** coated materials. **(F)** Log reduction of planktonic cells at 24 and 48 h. Error bars represent 95% CI.

Tryptic soy broth (TSB)-grown biofilm and planktonic cell densities of *P. aeruginosa* for uncoated materials, coated materials, and log reductions are presented in Figure 2. The coating significantly reduced biofilm accumulation of *P. aeruginosa* (99.999%–99.9999%) over 24 and 48 h on all four materials as compared to uncoated materials. Uncoated and coated Teflon allowed the most *P. aeruginosa* biofilm accumulation at 24 and 48 h. Uncoated titanium and coated Inconel fostered the least accumulation. In TSB, the coating performed similarly well across all four materials and at each time point. Reactor broth was sampled at the start of continuous flow and at later time points to measure planktonic cells (Figures 2D–F). Coated materials in the reactor with TSB significantly decreased *P. aeruginosa* planktonic cell density throughout the experiment. The data in Figures 2D–F show the cell density at the time flow to the reactor was turned on (0 h) and after 24 and 48 h of operation as well as the density of the initial *P. aeruginosa* inoculum. The biocidal property of the coating had a strong negative effect on the *P. aeruginosa* planktonic cell numbers even at the start of flow to the reactor (Figure 2E). After 24 or 48 h of operation with coated materials, *P. aeruginosa* reactors revealed reduction of 99.9%–99.99% in planktonic colony counts when compared to uncoated materials.

**Figure 3:**
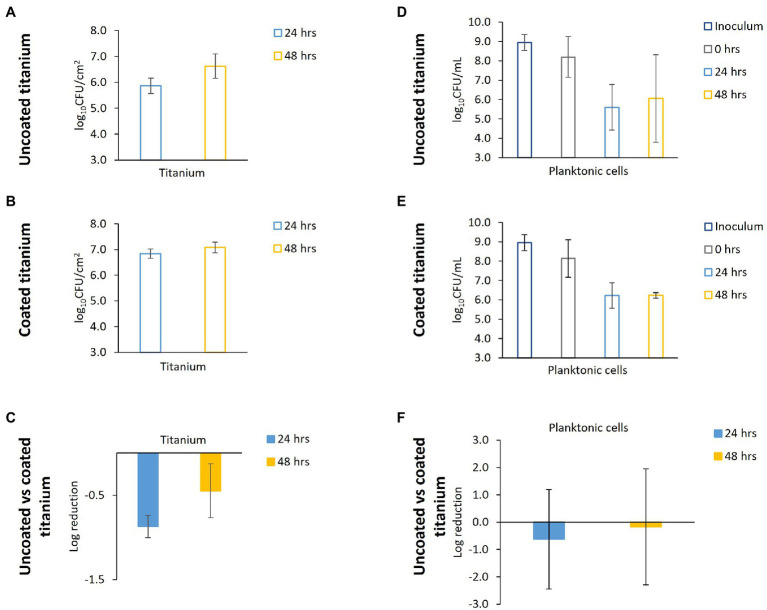
Potato dextrose broth (PDB)-grown *P. aeruginosa* biofilm accumulation and planktonic cell density. PDB-grown *P. aeruginosa* biofilm cell densities at 24 and 48 h after start of continuous flow on **(A)** uncoated and **(B)** coated titanium. **(C)** Log reduction of TSB-grown biofilm accumulation on titanium at 24 and 48 h. Inoculum and planktonic cell densities at 0, 24, and 48 h after start of continuous flow for reactors with **(D)** uncoated and **(E)** coated titanium. **(F)** Log reduction of planktonic cells at 24 and 48 h. Negative log reductions indicate the coating resulted in higher cell densities. Error bars represent 95% CI.

The potato dextrose broth (PDB)-grown biofilm and planktonic cell densities for uncoated Titanium, coated Titanium, and log reductions are presented in Figures 3A–C. In this system, the coating does not reduce biofilm accumulation. The log reduction after 24 h was −0.87, indicating greater biofilm accumulation on the coated titanium than on the uncoated titanium. The log reduction was less negative after 48 h (−0.45) but still indicated increased accumulation on the coated titanium. This trend was mirrored by the planktonic cell density (Figures 3D–F) in the reactor broth at 24 and 48 h after start of continuous flow where log reductions were −0.63 and −0.17, respectively.

### SEM Analysis of CDC Bioreactor Materials

**Figure 4:**
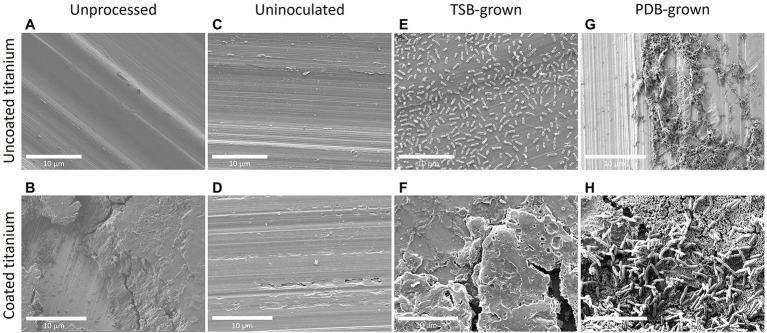
Scanning electron microscopy (SEM) imaging of *P. aeruginosa* biofilm formation on titanium after 48 h. Images **(A,B)** represent uncoated and coated titanium that were unprocessed (not exposed to the reactor, bacteria, or SEM preparation steps). Images **(C,D)** represent uncoated and coated titanium that were not exposed to the reactor or bacteria but went through the SEM preparation steps at the JPL. Images **(E,F)** represent uncoated and coated titanium that came from a reactor that utilized TSB medium while **(G,H)** came from a reactor with PDB. Biofilms on uncoated materials hosted numerous cells with small amounts of dried EPS attaching the cells to each other and to the surface.

**Figure 5:**
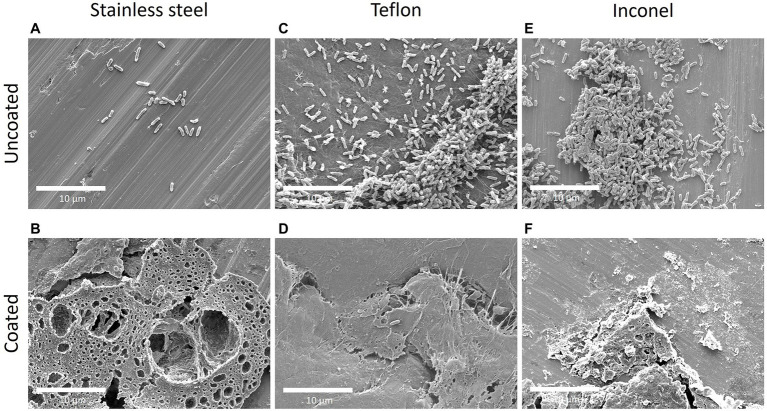
SEM imaging of TSB-grown *P. aeruginosa* biofilm on stainless steel, Teflon, and Inconel after 48 h. Images **(A,B)** represent uncoated and coated stainless steel; **(C,D)** represent uncoated and coated Teflon; and **(E,F)** represent uncoated and coated Inconel. All biofilms were grown in reactors with TSB as nutrient medium. The coated stainless steel demonstrates the presence of coating and coating debris.

SEM images of coated and uncoated coupons were taken throughout the experiments, including unexposed coupons direct from manufacturing, uninoculated coupons exposed to the SEM preservation and preparation process, and those exposed to *P. aeruginosa*. SEM images of Titanium coupons are presented in Figure 4. Stainless steel, Teflon, and Inconel SEM images are in Figure 5. Coupons not exposed to the SEM preservation and preparation process were imaged to determine potential effects of the preparation process on the surfaces (Figures 4A,B; Supplementary Figures 1A1–2, B1–2, C1–2). Images of uninoculated materials present smooth surfaces relatively free of debris and deformity (Figures 4C,D; Supplementary Figures 1A3–4, B3–4, C3–4), though it appears the coating detached in some areas (Supplementary Figure 1A4). Striations and concentric rings were observed showing evidence of the manufacturing process.

SEM analysis of CDC bioreactor *P. aeruginosa* grown in TSB for 48 h demonstrated production of extracellular polymeric substances (EPS) and the formation of cell agglomerations on uncoated surfaces. This biofilm morphology was observed across all uncoated materials (Figures 4E, 5C,E) except for stainless steel, which had a much lower cell density (Figure 5A). Coated materials had substantially decreased cell abundance across all material types, with only a few isolated attached cells identified (Figures 4F, 5B,D,F).

SEM images of biofilms grown in PDB for 48 h present similar characteristics to the TSB-grown biofilms. However, there were large biofilm agglomerations present on coated surfaces as well as uncoated surfaces. The titanium is clearly visible on the uncoated coupon in addition to cells clumped together (Figure 4G). On the coated coupon, the titanium is not visible but there is evidence of the coating cracking and lifting off of the surface beneath the attached cells (Figure 4H).

## Discussion

Along with testing a variety of materials simultaneously, CDC bioreactors grow biofilms in a high shear environment to allow for robust biofilm growth. The coating selected here previously showed high antimicrobial efficacy in hospital settings (Perez et al., 2015; Fitton et al., 2017) and exhibited grade 1 toxicity; indicating the coating is below the grade 2 cytotoxic agent threshold, and thus is safe for human contact (Baxa et al., 2011). Since this is a proprietary commercial product, the composition is unfortunately unavailable.

The Environmental Protection Agency details performance test guidelines for antimicrobials of varying types and applications (Environmental Protection Agency, 2015). These guidelines include a 3-log reduction and served as a reference point to evaluate coating performance in tests presented here. The present study included direct microorganism contact with surfaces for 48–72 h (including 24 h spent in batch phase) that still exhibited a 5–6 log reduction of TSB-grown *P. aeruginosa* biofilm on coated surfaces compared to uncoated controls on all four ISS materials (Figure 2C). The mixed effects revealed by the ANOVA show that the sampling time, experiment number (replicate), and material type had no effect on the log reduction. The log reductions from 24 to 48 h were not significantly different. There was no influence from the experiment number (replicate) on the log reduction. Lastly, the log reductions from material to material were similar, meaning the coating performed similarly across all materials. As the material did not affect the efficacy of the coating, only one material was chosen for the PDB tests. Titanium was chosen as there was the greatest abundance of coated and uncoated coupons remaining from previous experiments. Unlike the TSB-grown biofilms, the PDB-grown *P. aeruginosa* biofilm accumulation was not reduced by the antimicrobial coating (Figure 3C). PDB is essentially made by boiling potatoes, dehydrating the leached nutrients, and supplementing with dextrose. It is possible that the starches found in potatoes interact with the coating in a detrimental manner. The starches could be forming a protective layer on top of the coating, preventing the biocidal action (“carbon-chain lances”) from inhibiting biofilm accumulation in PDB as seen by the negative log reductions in Figure 3C. It is possible that *P. aeruginosa* forms more resistant biofilms in PDB compared to TSB and thus, the coating did not reduce biofilm accumulation. TSB and PDB have different pH (7.1–7.5 and –5.1; respectively), and although not specifically addressed by the vendor as being an issue, pH may alter the effectiveness of the surface coating’s “carbon-chain lances.” Additionally, it has been demonstrated that changes in pH can alter biofilm forming ability (Jones et al., 2015). We used the TSB and PDB growth media at their native pH as to avoid the addition of buffering salts, which may have compounded the issue by interacting with the surface coatings and causing differential biocidal activity. A minor limitation of this study is that we did not perform a full test matrix across all pH and media types to characterize the changes within the biofilms on uncoated and coated surfaces; however, this approach would have been outside the scope of this paper, and our results that the antimicrobial surface coating is as drastically impacted by media type (regardless of pH) are a significant finding.

Extended tests are needed to determine long-term antimicrobial coating efficacy in TSB. In addition, influence of inoculum cell density on antimicrobial coating effectiveness should be tested. Starting cell densities tested here were 10^5^–10^6^ CFU/ml. Coatings have been shown to reduce biofilm accumulation on many surfaces; however, often once a number of microbes attach to a surface, the coating quickly becomes ineffective due to establishment of a conditioning layer (Zea et al., 2020). This may help explain the increase in *P. aeruginosa* CFU/cm^2^ observed on coated materials from 24 to 48 h. The SEM results demonstrate a similar pattern of biofilm abundance across material types and coatings as CFU analysis for both TSB and PDB-grown biofilms (Figures 4, 5).

The coating had very different effects on viable *P. aeruginosa* biofilms grown in TSB and PDB; largely decreasing *P. aeruginosa* CFU in TSB with little effect on CFU in PDB. The antimicrobial coating is a charged substance that imparts a positive charge to the substratum surface (ISS materials) *via* a nitrogen compound which attracts negatively charged microbes. The biocidal coating then works by penetrating and damaging cell envelopes with positively charged nitrogen on a carbon-chain “lance.” This penetration can disrupt cellular proton motive force and rupture cell membranes leading to lysis and death (Murray et al., 2017). *P. aeruginosa* is a Gram-negative bacterium and its cell envelope consists of an inner and outer phospholipid bilayer separated by a thin peptidoglycan layer measuring 20–30 nm in total thickness (Salton and Kim, 1996). In the TSB system, the coating was likely uninhibited, thus resulting in penetration of the cell membrane and consequent biocidal effects from the coating. However, in the PDB system, it is possible that the starch from the potatoes created a protective carpet that the bacteria were able to attach to without being lanced by the coating’s carbon chains.

Although CDC bioreactors create conditions that mimic biofilm inducing environments, they cannot produce an identical spaceflight environment including microgravity, increased radiation, and CO_2_ concentration (Huang et al., 2018). They additionally cannot model spaceflight environments not fully submerged in flowing water where biofilm formation might still be of concern. Such limitations can be addressed with future experiments using different methods to grow biofilms, including simulated microgravity reactors and intermittent submersion of test materials (filling and draining of reactors). Studies such as this one aim to gain an understanding of how antibiofilm regimens work in simplified normal gravity systems to then move promising regimens into more complicated systems that closely mimic the conditions on the ISS. The development of suitable antimicrobial coatings in combination with human-friendly biocidal compounds is warranted. When designing antibiofilm regimens, it is important to assess biofilm formation in various environments. This study provides critical insight into the importance of testing an antibiofilm strategy in differing media as they could alter the efficacy of the strategy.

## Conclusion

While the coating is promising for reducing *P. aeruginosa* biofilm accumulation in TSB, experiments in PDB revealed an ineffective coating. It is hypothesized that the starch from PDB interfered with the coating, rendering it ineffective. Additionally, these experiments included high nutrient medium designed to grow robust biofilms on short time scales. As demonstrated in this study, it is possible that the experiments could yield different results using media more similar in chemical composition and nutrient availability to that found on the ISS. Systems like those where the coating would be implemented should be tested. Moreover, the combination of surface coated materials (as shown here) along with non-toxic biocides should be tested. While surface coatings are not the sole solution to reducing biofilm growth in long-term spaceflight, coatings will likely be an integral part of the eventual solution to mitigate biofilm accumulation in current and future crewed spacecraft.

## Data Availability Statement

The raw data supporting the conclusions of this article will be made available by the authors, without undue reservation.

## Author Contributions

KV and BP designed the study, evaluated results, discussed the results, and helped to write manuscript. MKM conducted CDC bioreactor assays, performed microscopy, generated figures, and drafted the manuscript. CWP procured and prepared materials (with and without coating), performed microscopy, worked with MKM, and drafted the manuscript. All authors contributed to the article and approved the submitted version.

## Funding

This research was supported by the ISS Advanced Exploration System Life Support System program funded to JPL and supported post-doctoral fellowship to CWP. The funders had no role in study design, data collection and interpretation, the writing of the manuscript, or the decision to submit the work for publication. No funding was obtained from the company that developed and provided the coating. MSU support for a graduate fellowship to MKM was provided by JPL under subcontract #1635633.

## Author Disclaimer

This manuscript was prepared as an account of work sponsored by NASA, an agency of the US Government. The US Government, NASA, California Institute of Technology, Jet Propulsion Laboratory, and their employees make no warranty, expressed or implied, or assume any liability or responsibility for the accuracy, completeness, or usefulness of information, apparatus, product, or process disclosed in this manuscript, or represents that its use would not infringe upon privately held rights. The use of, and references to any commercial product, process, or service does not necessarily constitute or imply endorsement, recommendation, or favoring by the US Government, NASA, California Institute of Technology, or Jet Propulsion Laboratory. Views and opinions presented herein by the authors of this manuscript do not necessarily reflect those of the US Government, NASA, California Institute of Technology, or Jet Propulsion Laboratory, and shall not be used for advertisements or product endorsements.

## Conflict of Interest

The authors declare that the research was conducted in the absence of any commercial or financial relationships that could be construed as a potential conflict of interest.

## Publisher’s Note

All claims expressed in this article are solely those of the authors and do not necessarily represent those of their affiliated organizations, or those of the publisher, the editors and the reviewers. Any product that may be evaluated in this article, or claim that may be made by its manufacturer, is not guaranteed or endorsed by the publisher.

## Supplementary Material

Supplementary Figure 1 Additional SEM images. SEM images of **(A)** Stainless steel, **(B)** Teflon, and **(C)** Inconel. Images **(1)** and **(2)** for each block represent uncoated and coated materials that were not exposed to the reactor or microorganisms and did not go through any SEM preparation steps. Images **(3)** and **(4)** represent uncoated and coated materials that were not exposed to the reactor or microorganisms but went through the SEM preparation steps.
