# Supplementary figures and images for: Antimicrobial Coating Efficacy for Prevention of Pseudomonas aeruginosa Biofilm Growth on ISS Water System Materials
Source: Front Microbiol. 2022 Apr 7;13:874236. doi: 10.3389/fmicb.2022.874236 (PMC9022025; doi:10.3389/fmicb.2022.874236)

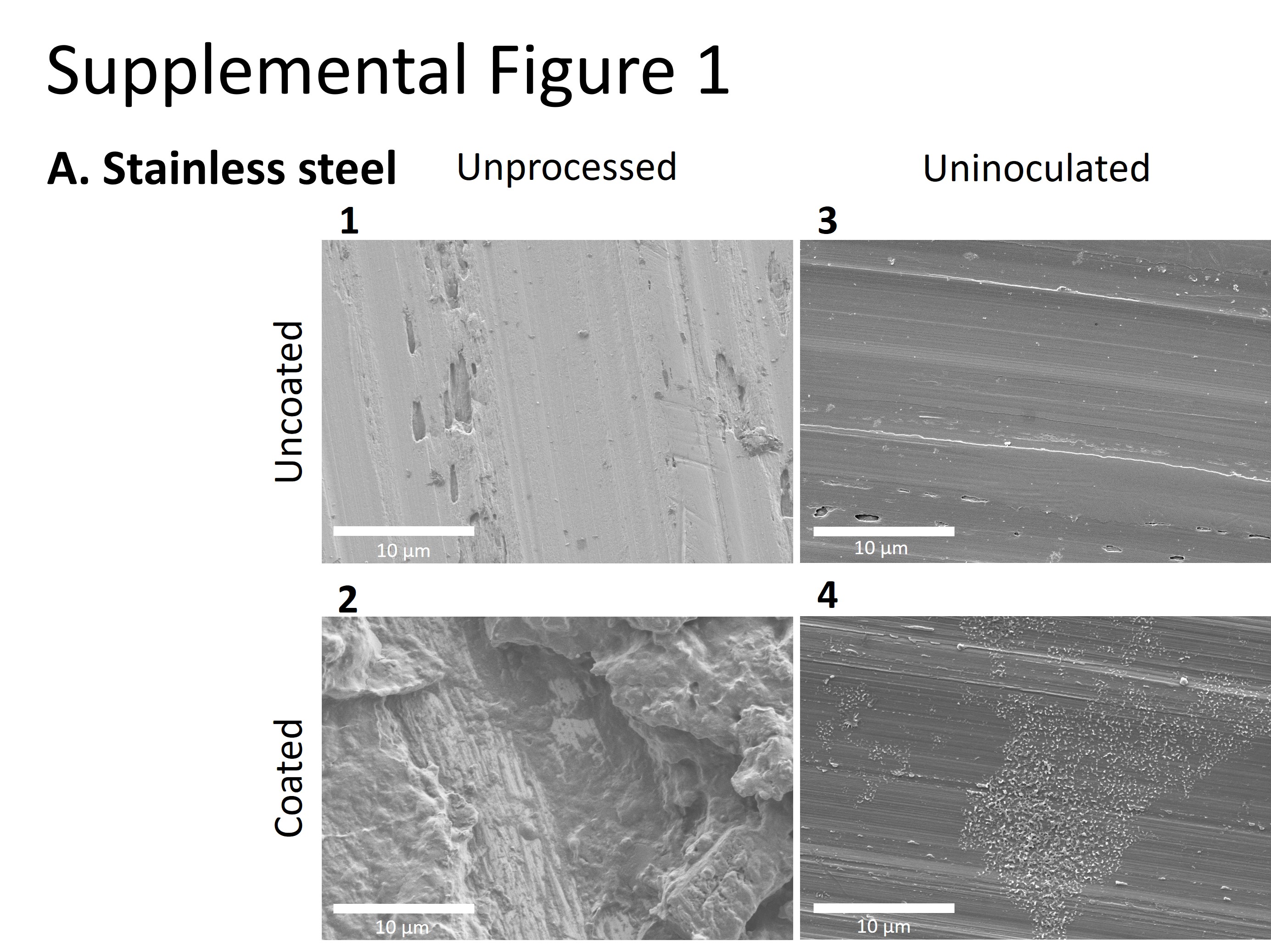

Supplement: Supplementary Figure 1 — Additional SEM images. SEM images of (A) Stainless steel, (B) Teflon, and (C) Inconel. Images (1) and (2) for each block represent uncoated and coated materials that were not exposed to the reactor or microorganisms and did not go through any SEM preparation steps. Images (3) and (4) represent uncoated and coated materials that were not exposed to the reactor or microorganisms but went through the SEM preparation steps. [file Image_1.JPEG]

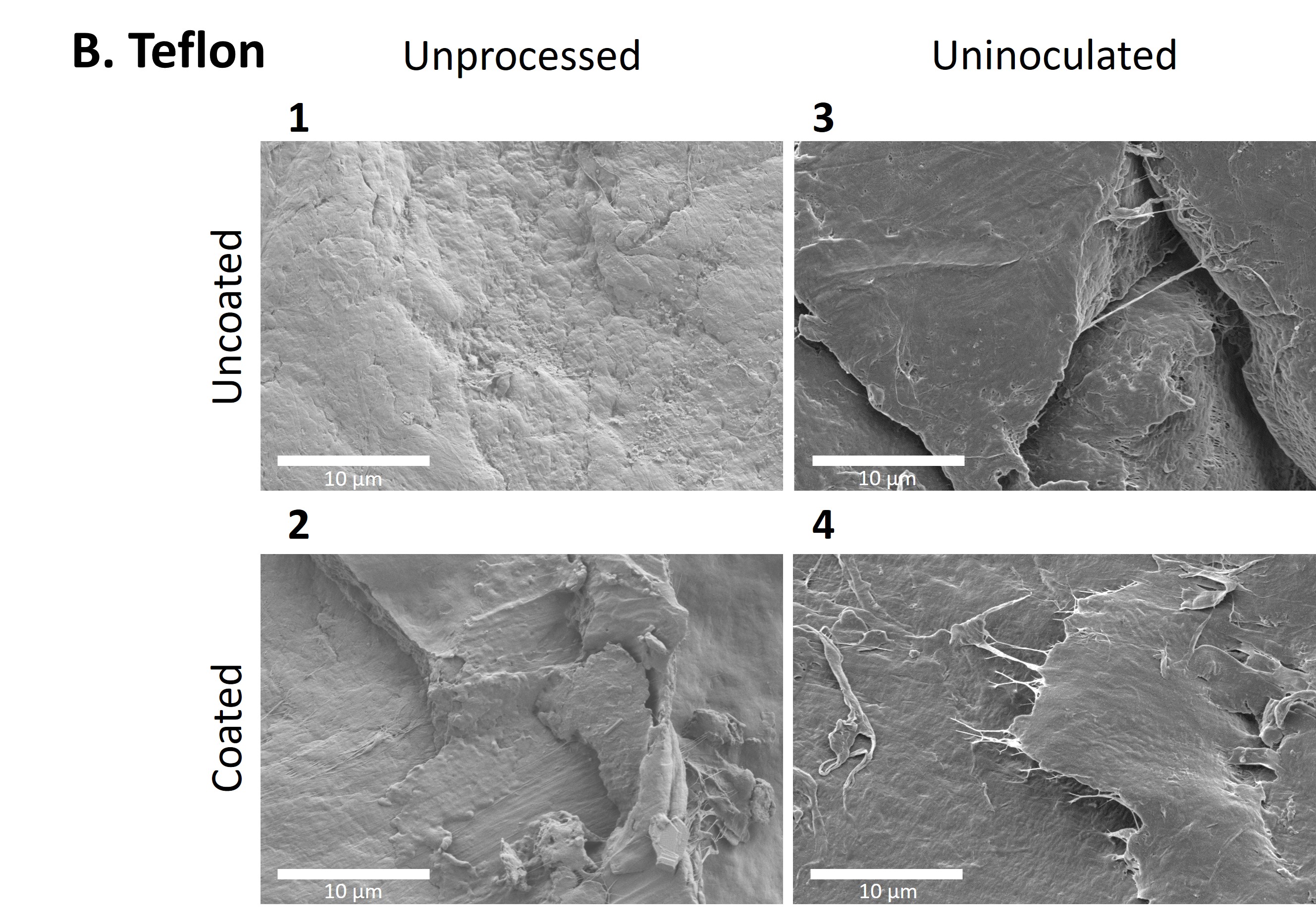

Supplement: Supplementary file 2 [file Image_2.JPEG]

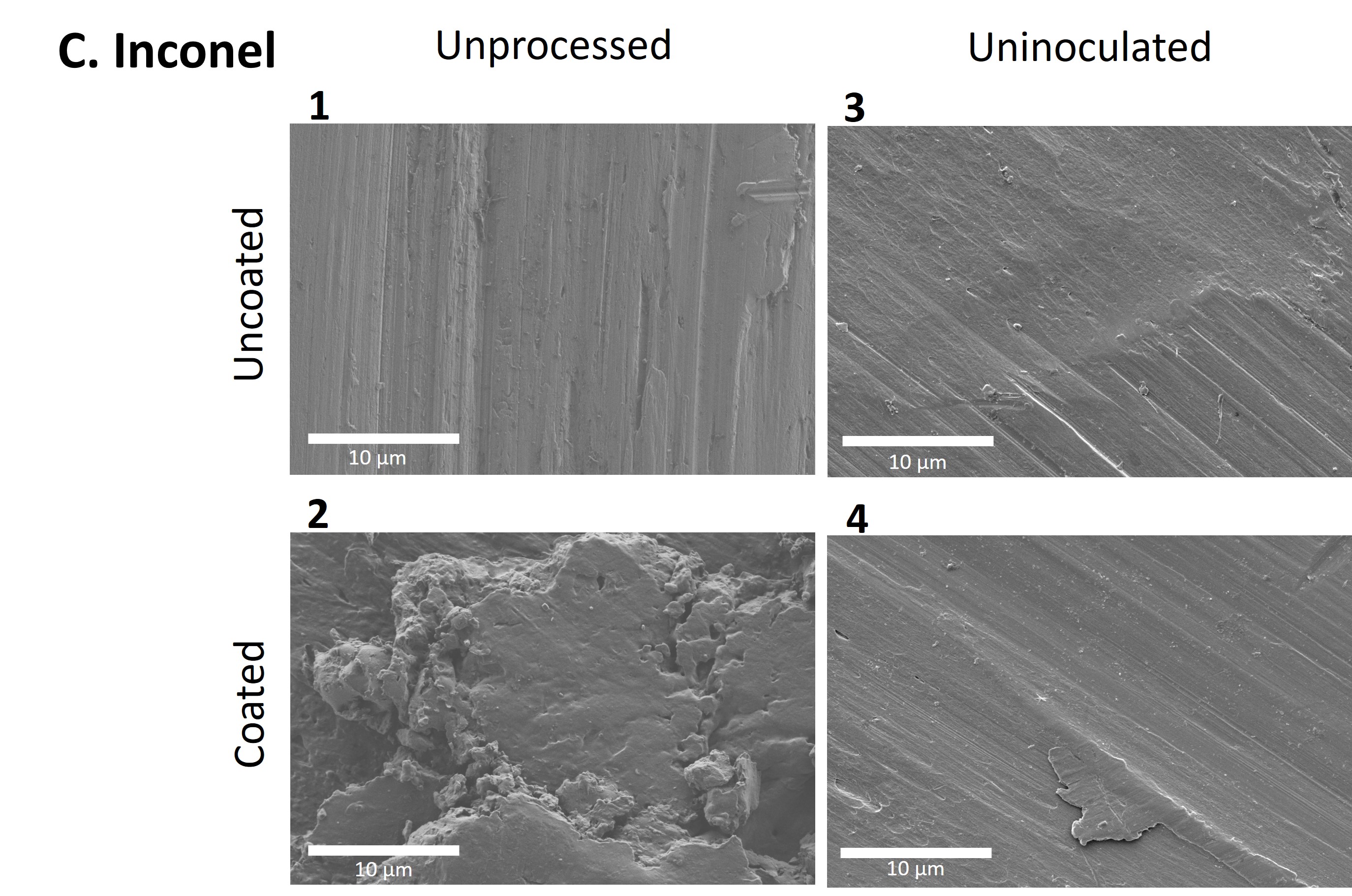

Supplement: Supplementary file 3 [file Image_3.JPEG]
